# Enablers and barriers to engaging under-served groups in research: Survey of the United Kingdom research professional’s views

**DOI:** 10.3310/nihropenres.13434.1

**Published:** 2023-07-26

**Authors:** Dorothy Coe, Theophile Bigirumurame, Meera Burgess, John Rouse, Caroline Wroe

**Affiliations:** 1 Newcastle Upon Tyne Hospitals NHS Foundation Trust, Newcastle, England, UK; 2 Population Health Sciences Institute, Newcastle University, Newcastle upon Tyne, England, UK; 3 Clinical Research Network Coordinating Centre, National Institute for Health and Care Research, NIHR, UK; 4 Clinical Research Network North East and North Cumbria, National Institute for Health and Care Research, Regent Point, Newcastle, UK

**Keywords:** Research inclusion, research participation, under-served, underrepresented, enablers, barriers.

## Abstract

**Background**

There is a known lack of diversity in research subject populations. This impacts on the generalisability of findings and effects clinician prescribing. In the United Kingdom those who are underrepresented are thought of as under-served by the research community. They are commonly those affected by health inequality and disparity. Works illustrate the complexity of the issue covering demographic, social, economic and illness factors which contribute to being under-served and strategies to improve engagement. The survey aim was to explore the views of research professionals in the UK.

**Methods**

Research professionals were invited to complete an online survey. The findings were analysed using statistical and qualitative methods to identify the enablers and barriers to engagement of under-served groups in research. Descriptive statistics were utilised with associations compared univariately using chi-square test and logistic regression for multivariable analysis.

**Results**

A total of 945 completed responses were received. Those identified as under-served agrees with the previous literature with a broader descriptor of ethnic and cultural minorities and the addition of adolescents and young adults. Language and literacy skills have the most impact on being under-served. Only 13% of respondents said they felt equipped to meet the needs of the under-served. The main strategy to increase diversity was community engagement and the movement of research delivery into the community. The largest barriers were funding and time. Training needs identified were linked to community engagement, cultural competence and consent processes.

**Conclusions**

The UK findings reflect the previous USA literature. Adolescents and young people are added to the groups identified as under-served. Enablers included community outreach and improvement to communication. Barriers were time, funding, organisational processes and lack of focus. Issues were identified with translation and interpretation services. Training requirements focus on methodologies and methods to engage and the consent of those from under-served groups.

## Plain language summary

Research study participants do not reflect the general population. This means that findings from research cannot be reliably transferred to parts of the general population. The people missing are termed under-served or underrepresented. They come from groups who have unequal access to health care and have poorer health. There are different reasons why people may not take part in research and many ways to get more of them to take part.

This project asked the people who work in research who they thought were missing from research studies. It asked, what would make it easier for people to take part and what got in the way. They were invited to write their thoughts in a questionnaire found on the internet. Answers were then looked at using tests to find out the number of people who said the same or different things. Other answers were looked at to find out their views and opinions.

A total of 945 people filled in the questionnaire. They thought the biggest group of people who were missing from research populations were from minority social and cultural groups. They thought teenagers and young adults were missing from research. When asked why people didn’t take part the biggest problem was with making research easy to understand. Only 13 out of every 100 people who filled in the survey thought they were able to help those who were missing. It was suggested the best way to improve the number of different people taking part in research was to carry out as much research as possible in the community. The biggest thing that stopped those working in research helping was funding and time. They wanted to find out how to move research into the community, how to help different cultural groups and aid those with certain medical conditions.

## Introduction

There is a lack of diversity in the participants of research studies. In the United Kingdom (UK) the groups thought of as underrepresented are termed under-served. The definition being: ‘A group that is less well represented in research than would be desirable from population prevalence and healthcare burden’ ^1^. Commonly these groups are disproportionally affected by ill health, live in poverty and have associated health inequality and health disparity ^2, 3^. Their lack of representation reduces the validity and reliability of research findings and inhibits generalisability ^4^. This results in interventions and treatments being approved with limited evidence for their use in the under-served populations ^5, 6^. The lack of evidence affects the willingness of clinicians to prescribe, limiting treatment options and continuing the cycle of health disparity and inequality ^7^.

In recent years there has been a move to improve the representation of under-served groups in research. There is a large body of work, mainly from the United States of America, where the preferred term is ‘underrepresented’. In the UK the National Institute for Health and Care Research (NIHR) funds, enables and delivers research; it has funded and completed two major projects aimed at engaging potential research participants ^7, 8^. NIHR INCLUDE ^7^ specifically focused on those who may be under-served, involving them directly in producing the definition above. Prior to this there was no formal definition of what constituted an under-served or underrepresented group, the terms being context specific and used interchangeably. Very recently the all-party parliamentary group on medical research published a report on health disparities and why medical research is a crucial tool for change ^9^. This report included a section on recruiting a more diverse range of participants to clinical research.

Illustrating the multifaceted complex and interrelated nature of the concept, the previous works link the terms to one or more of the following: a demographic identifier such as age ^5, 10^ or ethnicity ^11, 12^, a specific disease such as cancer ^13, 14^, or a strategy aimed at engagement ^15, 16^. Many narrow the focus to engagement within a named group and a specific disease ^17, 18^ and others narrow further to named group, named disease and named engagement intervention ^19, 20^. Engagement strategies fall into five main areas: changes to research methods and methodologies ^21– 23^, organisational initiatives ^7, 24, 25^, use of technology ^19, 26^, enhancing trust ^27, 28^ and finally changes to reporting and publication ^29, 30^.

This article forms part of the output from a cross-UK NIHR programme board with specific emphasis on engaging under-served communities in research. It reports on a survey which explored the views of research professionals. The survey covered: who they felt were the under-served, what factors led to being under-served, what changes promoted inclusivity, what barriers exist and what additional training and resources were required to aid engagement activity.

## Methods

### Patient and Public Involvement

There was no patient or public involvement in this project as it was aimed at professionals working in research.

### Ethics and consent

The NIHR under-served programme board agreed that ethical approval was not required for this research, as the survey was deemed service improvement. Participants’ consent was presumed by the active choice of clicking the link to the survey and completing the questions. Responses were anonymous. The survey data were stored on password protected devices and only accessed by the research team. Free text comments were reviewed prior to analysis and any identifying information removed or coded.

### Survey development

An online survey was designed and delivered in smartsurvey.co.uk ^31^. It was produced following a focussed review of the literature. The review was completed between July and September 2021 using the terms ‘under-served’ and ‘underrepresented’. PubMed database was utilised, and the search was limited to articles produced between 2018 and 2021, where full-text or abstracts were available. All article types were included and the biographies of each reviewed. A total of 62 articles were identified and reviewed for relevance. After the initial assessment, 47 articles formed the focussed review.

The subsequent survey was piloted and peer reviewed by six members of the NIHR under-served programme board. Feedback from peers was discussed and acted upon and there was one further round of revisions. A copy of the survey is available ^32^.

### Recruitment and data collection

The survey was advertised through an NIHR communications package. It was circulated to all local clinical research network communications leads and partner organisations with a request to share. It was promoted via regional NIHR newsletters and the Clinical Research Network national newsletter. Further promotion appeared on Twitter and LinkedIn. As an example, in the North East and North Cumbria CRN, the request to share was actioned by the communication lead. This led to the direct emailing of research and development managers, speciality groups leads and core team delivery staff which totalled 89 direct emails. The recruitment aim was to reach those working in the public sector of the UK who were involved in research delivery. There was no defined target sample and there were no specific exclusions.

The survey commenced with an introductory statement and link to the NIHR INCLUDE project. It was split into four sections totalling 19 questions. The first section of nine questions collected demographic and role information. This was followed by one question regarding specialism. The next seven questions were titled ‘Barriers and enablers’ and gathered information on who are the under-served, factors impacting on being under-served and strategies to improve research activity to better engage the under-served. The final two questions allowed respondents to indicate if they wanted to make direct contact with research team and leave their contact details. Question types included closed, open, ranking and four free text response questions. Only complete data for each section was analysed.

### Analysis

Statistical analysis was performed using R software ^33^. Descriptive statistics were utilised with associations compared using chi-square test. To test relationships between categorical variables statistical significance was deemed as p. value of <0.05. Qualitative analysis of the free text responses was completed using a thematic approach ^34^. The analysis followed three steps. Initially the free-text comments were read to familiarise and anonymise. The comments were then coded and descriptive themes generated. The themes were guided by the findings from the focussed literature review. Finally, the descriptive themes were reviewed to generate an overall viewpoint. ^34, 35^. Analysis was aided by Microsoft Excel, topic models ^36^ and tidytext ^37^ packages.

### Reflexivity

The process was verified for trustworthiness by the core team. Reflexivity was demonstrated by acknowledging the prior views and knowledge of the research team. Themes were guided by the previous literature, however differing views and information were explicitly sought out. Data were coded by different individuals and data analysis clinics held to reflect on codes and discuss initial theorising ^34^.

The authors have experience of survey research (DC, CW, JR), statistical (TB) and qualitative analysis (DC). CW was responsible for the original idea, CW, JR and DC formulated the questions and JR built the survey. MSB has expertise in equality and diversity and aided the qualitative analysis with DC. The study is reported in line with CROSS ^38^ (Checklist for Reporting Survey Studies), STROBE (STrengthening the Reporting of OBservational studies: cross -sectional studies) ^39^ and SRQR (Standards for Reporting Qualitative Research) ^40^ guidelines.

## Results

### Demographics

Data was collected from 945 respondents who confirmed they were happy to complete the survey ^32^. Responses were received from all geographical areas of the UK. The largest number came from the North East and North Cumbria (n=111, 12.4%). The age, sex and ethnicity profiles reflect that of the UK’s National Health Service (NHS) with 72% (n=660) identifying as female, 82% (n= 748) white and over 30% (n=275) in the 45–54 years age group ^41^. Respondents self-identified their sex, the options being male, female, prefer not to say and other (please specify). All components of the NIHR infrastructure and Clinical Specialties in the NHS were represented. To identify any differences in enablers and barriers across clinical settings, sub-set analysis via specialism was completed. These were: hospital-based specialties (n=323, female 222, male 91), mental health (n=124, female 82, male 38), and primary care (n=118, female 90, male 24), including public health and community. These three sub-sets total 60% (n=565) of the total responses.

### Place of work and role

The commonest area worked in was secondary care (41%, n= 371, female 259, male 102), followed by research higher education institutions (39%, n=355, female 242, male 97). The least cited place of work was the ambulance service (0.3%, n=3). When asked, 75% (n=587, female 290, male 153) of those who responded stated they were involved in identifying participants for research studies, with 69% (n=624, female 442, male 164) saying they had direct contact with research participants and finally 54% (n=491, female 353, male 128) declared they were involved in consenting participants for research studies.

### Who are the under-served?

The respondents were asked to identify who they felt were under-served in their area. They were given five open text boxes to insert a descriptor. They were not asked to rank the five descriptors. In total 2782 responses were made across the five open text boxes. The responses were collapsed into categories. Table 1 illustrates the top seven categories and the total responses for each. These findings broadly reflect the previous literature. The most cited group was ethnic and cultural minorities, followed by those deemed as having a lower socioeconomic status, then those where a given age was used as an identifier. In the category where age was used as an identifier, ‘adolescents/young adults’ were put forward as a group not previously identified. The ethnic and cultural minority group contained a broader descriptor than found in the literature which focussed on Black and Hispanic groups.

**Table 1.** Who are the under-served in your area? The respondents were asked to identify who they felt were under-served in their area. Table one illustrates the top seven categories and the total responses for each. These findings broadly reflect the previous literature. Subset analysis by specialism reveals that the hospital-based group placed ‘condition specific’ (9%, n=76) above ‘mental health’ (6%, n=52), with those specialising in mental health putting ‘mental health’ in second place (18%, n=79) followed by ‘lower socioeconomic status’ (14%, n=62) ‘learning/intellectual difficulty/disability’ (11%, n=48) and age related at 8% (n=36).

Description	Total number across the five open text boxes.	% of total responses (n=2782)	% in hospital- based specialism (n=887)	% in mental health specialism (n=450)	% in primary care specialism (n=415)
Ethnic and cultural minorities.	862	31%	34% (n=305)	26% (n=119)	33% (n=136)
Lower socioeconomic status	514	18%	17% (n=151)	14% (n=62)	22% (n=93)
Age related. (Older, younger, adolescent)	304	11%	10% (n=92)	8% (n=36)	10% (n=42)
Mental health, dementia and neurodegenerative diseases	213	8%	6% (n=52)	18% (n=79)	7% (n=30)
Condition specific (excluding mental health, dementia and neurodegenerative diseases)	143	5%	9% (n=76)	2% (n=10)	3% (n=11)
Learning/intellectual difficulty/ disability (including ADHD and autism)	131	5%	4% (n=33)	11% (n=48)	3% (n=13)
Access (rurality, workers, limited mobility, car-less) excludes digital access.	139	5%	4% (n=39)	4% (n=19)	6% (n=24)

Subset analysis by specialism reveals that the hospital-based group placed ‘condition specific’ (9%, n=76) above ‘mental health’ (6%, n=52), with those specialising in mental health putting ‘mental health’ in second place (18%, n=79) followed by ‘lower socioeconomic status’ (14%, n=62) ‘learning/intellectual difficulty/disability’ (11%, n=48) and age related at 8% (n=36). The primary care-based specialisms elevated ‘access’ above ‘condition specific’ (p=0.038).

### What impacts on being under-served?

**Figure 1.**
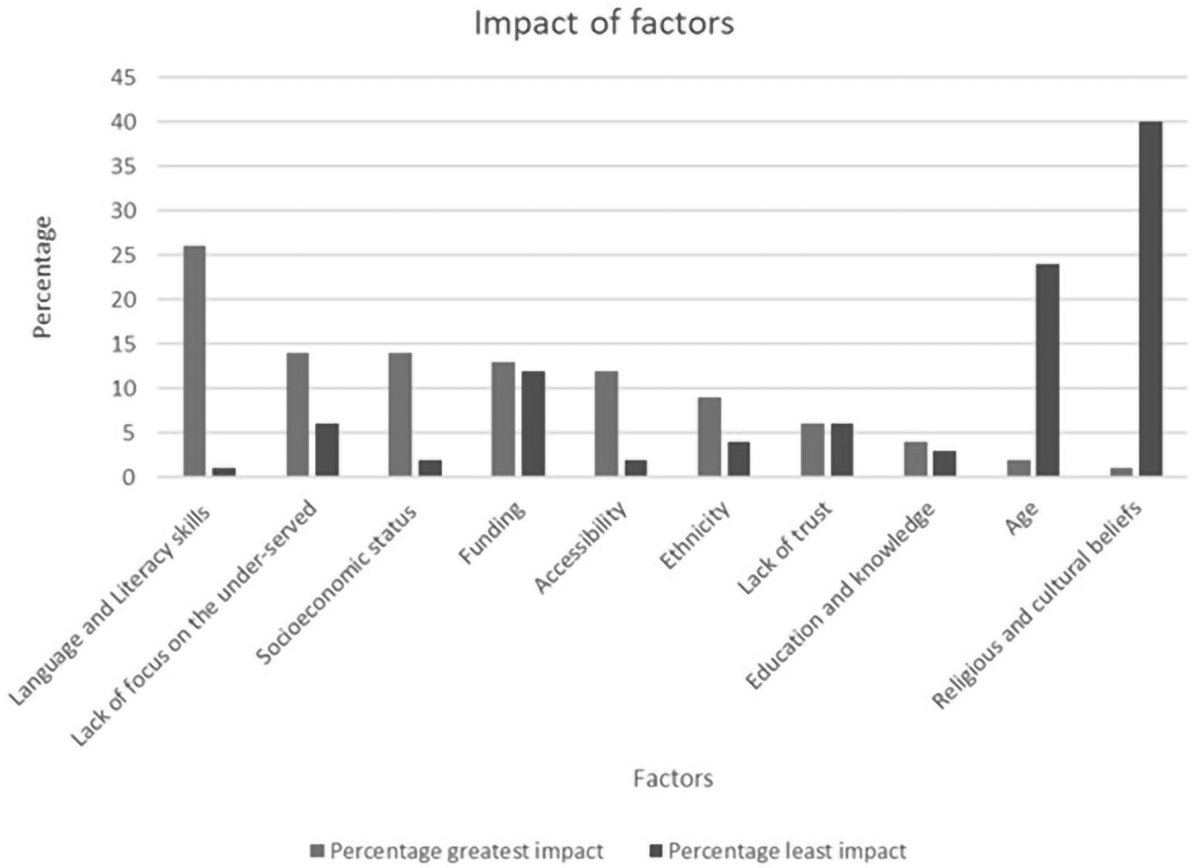
Ranking of 10 factors on degree of impact on being under-served. Respondents ranked 10 factors taken from the literature, in terms of how they impacted on being under-served. They ranked language and literacy skills as the most impactful, with the least impactful being religious and cultural beliefs. Figure 1 illustrates the ranking of factors.

Respondents ranked 10 factors taken from the literature, in terms of how they impacted on being under-served. They ranked language and literacy skills as the most impactful, with the least impactful being religious and cultural beliefs. Figure 1 illustrates the ranking of factors.

Sub-set analysis via specialism indicated statistically significant variation in greatest impact. Hospital-based specialisms (32%) and primary care specialists (30%) ranked language and literacy skills as the most impactful when compared to mental health 11% (p<0.0001). Mental health services ranked a lack of focus on the under-served as more significant: mental health 22%, primary care 15% and hospital-based 12% (p=0.04). In addition, significant differences were demonstrated for socioeconomic status, with this being ranked as a more significant factor in primary care research: primary care 22%, mental health 11% and hospital-based 10% (p=0.004).

### Addressing the needs of the under-served

A total of 824 (610 female, 214 male) responses were received from those who answered question 8 (Are you involved in identifying research participants? Yes/No) and question 15 (How equipped do you feel in dealing with the under-served? Yes/Partially/Not very well/No). Of the 538 (392 female, 146 male) who stated they were involved in identifying research participants, 12.3% (n=66, 44 female, 21 male) suggested they felt equipped to meet the needs of the under-served, with a further 48% stating they were partially equipped (n=258, 179 female, 79 male). Of those not involved directly in identifying research participants (n=286, 218 female, 68 male), 8% (n=22, 18 female, 4 male) said they felt equipped to meet the needs of the under-served (p=0.06). A significant difference is also seen in those who identify research participants who felt partially equipped (n=258, 179 female, 79 male) when compared to those who do not identify research participants (38%, n=108, 81 females and 27 males), p=0.006. As expected, those who identify potential research participants in their role felt more equipped compared to those who do not (p=0.0004), similarly staff taking consent from research participants felt more equipped than researchers who do not (p=0.0001). Those who consent research participants were more likely to feel partially equipped to address the needs of the under-served compared to those who do not consent (p=0.013), suggesting that face to face contact improves confidence in engaging the under-served.

### Qualitative analysis

Four free-text questions were analysed using a thematic approach. They covered positive innovations, barriers to innovations, challenges to engagement and further training requirements. From these responses enablers and barriers were identified and suggestions for training put forward.

### Enablers

Several enablers were recognised with comments suggesting strategies to enhance engagement. However, these were often accompanied by the barriers indicating why they could not be implemented. The most cited enabler was community outreach.

*Community based research - while not revolutionary - has proved to be highly effective when wanting to involve underserved groups.*
*Going into the community they live in, engaging at a time most convenient for communities (i.e. in day time in between school hours for young mothers) and in the mid-week evenings at 06:30pm/07:00pm start to allow those who may be working, holding quarterly/6-monthly events on Saturdays (whole day) with necessary support to do out-reach activities such that information can be shared and participations encouraged.*
*We are never sufficiently funded to do anything innovative. Funding only covers the most basic and efficient of recruitment (e.g., GP search and mailout) and despite our best efforts to make study documents appropriate, engaging and accessible they do not attract groups that we know are underserved.*

Community engagement was seen as beginning prior to any notion of conducting research. It included *genuine* participation by the community in setting its own research agenda via collaborative approaches and continued after the completion of any research project.

Genuine engagement - not "involvement" - leading to genuine co-creation of work that meets the needs of the community partners *as much* as it meets the needs of the researchers.

Linking to this was the use of Patient and Public Involvement and Engagement (PPIE) groups. These groups were highlighted as enabling, however there was a degree of scepticism about how representative they are of their group.

*Appropriate PPI (i.e. finding the right people to be involved in PPI not just some well-educated, often white person from a professional/semi-professional background). Research organisations should start thinking about recruiting community champions from under-served populations / communities. These should be influential people who can be able to help with ensuring participation of under-served populations. Use of community champions has been an effective approach by community-based organisations (CBO). So where research organisations are not able to directly recruit community champions, development of research partnerships with CBOs can be a very good way forward.*

Outreach also included the movement of research from a secondary care setting to the community. It was suggested methods and methodologies should focus on assisting the delivery of research in the community.

*Community Ageing Research 75+ cohort study - a national ageing research cohort study funded by* (organisation). (They) *use an opt-out approach to consent and have researchers who are proficient in community language skills. The opt-out approach supports engagement with older people with frailty, some of whom who find it problematic to actively respond to an invitation to participate in research as they may have mobility problems making it problematic (or simply burdensome) to provide a postal response using an opt-in approach. The approach also supports recruitment of people from minority ethnic groups, some of who speak dialects that do not have a written form, so require a proactive telephone call to discuss participation. Taking this approach,* (they) *have a recruitment rate of around 40% at all our sites, and 5% of study participants are from the south Asian community (15% in Bradford - consistent with the local demographic).*
*Need very intensive methods to recruit and then retain under served groups, e.g. provide transport, pay for travel, take research to the community.*

Further enablers were identified such as strategies to improve the accessibility of study documentation and the information presented in them. Suggestions were put forward covering multiple different modalities: translation and interpretation services, video, audio, digital platforms, social media, sign language and braille. Again, these suggestions were accompanied by the barriers to implementation.

*Using culturally relevant short drama/films to aid raising awareness in communities that do not typically engage with research. Having these in multiple languages to aids those who are not literate in their native language - this is expensive and typically the sector relies on family member to translate but this does not necessarily meet recommended guidelines for consent.*
*We have asked study teams if they can translate consent forms, information sheets, questionnaires etc. into different languages so that we include non-English speaking participants, however, all study teams have said they did not have enough money/funding to be able to do this and/or to cover the cost of any translators.*

Additional enablers to engaging a diverse research population were having a workforce that reflects a varied background and where possible the demographic profile of the under-served group. Recruitment via General Practice was thought to better reflect the demographic profile of the area and innovative ways to reimburse participants was also thought to enable engagement.

*More active engagement with community groups within diverse communities to support recruitment, developed study materials in formats more accessible to people from diverse communities and with low literacy levels. Trained and paid individuals from diverse communities to support recruitment to studies and as co-researchers - for example who speak the language or who are known to people in the community. Anything extra that involves more resource as this often isn't possible in the tight funding windows.*
*The benefits systems mean we can’t pay those who we most want to get involved or they are sanctioned. Funders and senior academics want involvement from all but it needs time, commitment, planning- can’t be overnight.*

At an organisational level it was suggested that study design and approvals processes should reflect those of the urgent public health studies carried out during the COVID-19 pandemic. Highlighted were changes in ethical approval, community engagement, consent processes, data collection methods and feedback of results.

*Covid has forced one of our studies to move from face to face follow up to remote follow up. This has lead to a higher recruitment rate as potential recruits are more willing to conduct the follow up at home or in their own time than physically attend a follow up appointment. This has improved accessibility.*
*Remote data collection (especially promoted by COVID requirements) improves access for some - but need a range of options to be really inclusive.*
*Electronic consent, (if appropriate). This speeds up the consent process and allows the patient to complete the consent at their own pace at home. This is particularly effective now many clinics are conducted remotely, so paper consenting is not possible.*
*In Covid research we used a lot of consultee consents for patients who were acutely unwell and we also had lots of ethnically diverse patients inpatients so our recruitment was much more diverse.*

Sub-set analysis indicated the most cited enabler from both primary care-based and mental health groups was community outreach, with hospital-based specialties citing changes to research design and methodology.

### Barriers

The most cited barrier to engaging under-served groups was time and funding; this was reflected across the whole dataset and the sub-sets of primary care and mental health specialties. The respondents were aware of the strategies to enhance engagement; however, these all required more time and funding, and that time and funding was not planned into project designs. They felt that the current systems and processes around grants/funding applications and ethical approval were obstructive and risk averse. Funding processes did not consider the planning needed to design studies that are co-produced with communities and approvals processes question innovative recruitment strategies and reimbursement of participants. Comments suggest a disconnect between policy and practical application.

*Time - it takes significantly longer to take someone whose first language is not English through a study.*
*The whole ethics process is not set up for considering the needs of this population.*
*Need NIHR to ensure there is funding to support this and to understand that there is a substantial time and resource commitment to undertaking research that addresses under-served populations.*
*We are required to develop patient information sheets that go against the advice of our patients and basically, we know that they aren't read but we churn them out because we don't get approval otherwise. If you have a non-English speak/illiterate person and they are faced with some of the approved documentation, it’s no wonder they won't even talk to you…. There is no point in trying to be even slightly inventive because you just know the REC* (Research Ethics Committee) *is going to be a barrier to you actually being able to use it.*

Barriers also included issues with communicating research to specific groups who may be under-served. Respondents highlighted problems with translation and interpretation services and how useful they are in the field of research.

*Across multiple studies, we have been limited in the number of non-English speakers that we have recruited due to a lack of access to translators. The issues we have faced around translation is a lack of availability of translators and a lack of funding for translation services.*
*Another issue is being dependent on a translator communicating in the way you want them to - I had experience of a situation where the translator deemed that catheterisation of a little girl meant she was no longer a virgin.*
*Paying for actual translators not having to rely on family (which can often mis-translate, negate details, or might prevent sensitive topics being explored).*

Their concerns were around availability when required, knowledge levels and personal views of the translator or interpreter. Cost of translating written materials and the time taken were also thought to be obstructive. These were the most cited barriers in the hospital-based specialism. There were also comments which suggested that while the use of digital platforms may for some enhance engagement, it conversely disenfranchises some others.

### Further information and training

Suggestions about additional information and training to aid researchers were diffuse. They included ways to conduct community engagement including methodologies, methods, inclusive recruitment strategies, for both participants and PPIE, funding applications and innovative reimbursement methods. Further general training was requested in cultural competence, taking in cultural awareness and sensitivity and general equality and diversity. Some specific under-served groups that were mentioned regarding engagement activities were the LGBTQi community, those with disability and the homeless. Finally, requests were made for training in consenting those where English is not their first language or their comprehension of English may be reduced, and those who may lack capacity.

*Cultural awareness training. I feel this would be helpful to me with regard to those of Pakistani and Roma ethnicity, and for the Deaf Community. Advice regarding how to identify and link up with local community groups, to find PPI contributors from underserved populations. Training in inclusive research study design, sharing best-practice and learning from the successes of other researchers.*
*Be trained in how to speak to those and consent those who have dementia or other mental problems in their older years…How to work with those whose English isn’t their first language.*
*How best to engage with such communities, build their respect and interest and to understand value of research.*

In summary, the findings from the survey generally reflect the literature in this area. Those working in the field of research feel only partially equipped to deal with engaging the under-served and request additional information, support and training. The responses overall are context specific and focussed around the respondent’s area of speciality. The main enabler to engaging the under-served is thought to be community outreach. However, there is a consistent reference to lack of time, funding, capacity and support to develop site and project level interventions to engage the under-served. Respondents were aware of numerous strategies to improve the diversity of research participants and there was a sense of divide between policy rhetoric and ability to practically apply.

## Discussion

The findings from the survey come from across the UK, with substantial representation from areas of known deprivation and rurality, both factors associated with being under-served ^2, 42^. This may reflect an increased awareness and engagement in these areas and indicate strategies are already being utilised to enhance representation.

The groups identified as being under-served reflect the literature and the previous NIHR work ^7, 8^. Whilst the literature from the USA focusses on Black and Hispanic communities as minority ethnic and cultural groups ^6, 43^ the UK findings suggest a wider descriptor including non-white, non-native English speakers, migrants, refugees, Roma and traveller communities. These groups plus those termed Black Asian and minority ethnic (BAME) were the largest group deemed under-served. Of note is that during the timespan of the survey the UK government recommended a move away from using BAME as a descriptor of ethnic minority groups ^44^. The second largest group identified as under-served were those who live in low socio-economic circumstances, including those who were digitally excluded, live in geographical areas known to have high levels of deprivation and those who are unemployed. This group was followed by those with an age descriptor attributed to them, the old, very old, young adults, adolescents, children and neonates. There is limited previous literature to suggest young adults or adolescents are under-served by the research community. This group was identified across numerous research specialities and is a finding worthy of further understanding.

In terms of why groups may be under-served, the impact of factors appeared to be cumulative in nature. Descriptors often cited more than one identifier, for instance an older person living in a care home, or a migrant living in a rural area. Therefore, the possibility of being under-served was increased as descriptors or impactful factors were attributed to a group. This and the differing order of who is felt to be under-served by each speciality group aptly demonstrate the context specific nature of the ‘under-served’ and the influence of workplace and specialism. When asked to rank the factors that contribute to being under-served, language and literacy skills were thought of as the most impactful. Whilst it was felt ethnic and cultural minority groups were the largest groups under-served, it appears the communication to groups is the most impactful. This ties into the barriers where the participant information sheet, which is required by all approved research studies, was seen as a barrier to inclusivity. It is suggested that the level of English comprehension required to understand research is an issue when trying to engage the under-served. This then links strongly to the suggestion than improvements are needed in communication and information accessibility. These include interpretation and translation, plus information being accessible across multiple different platforms and modalities. Examples were cited from the urgent public health work carried out through COVID-19, where PPIE and community engagement had been very successful ^45, 46^. This work was highlighted as community outreach in action and worthy of further study to explore application and impact.

While interpretation and translation were suggested as strategies to increase the diversity of research populations, there were also several issues with this approach, particularly from the hospital-based perspective. These issues were centred on availability of translated study documentation, of individual translators and interpreters when required, their understanding of research and the cultural norms and impartiality of the interpreters. Some of these have previously been reported ^47– 49^ however, there is limited empirical work in this area with central guidance covering the general health setting only ^50^. Translation of written study documentation is put forward as an inclusion strategy ^2^, however there is little information about how often this service is utilised and if it has a positive impact. Conversely it is known anecdotally that research sponsored by academic institutions often cites lack of funding as a barrier to translation. The areas of translation and interpretation are worthy of further investigation into the use of, and levels of satisfaction in these services, from both research professionals and participants perspectives. This may lead to a stronger rationale for their use or suggestions for different approaches.

The strongest enabler was community outreach. Recommendations are made to explore focussing research delivery outside of secondary care. It is known that engaging communities in identifying their own needs and requirements has many positive benefits ^51^. There are innovative strategies that allow interventional studies to be conducted wholly or partially in the community where the under-served are ^22, 43^. General practices are seen as providing access to local communities who reflect the demographic profile of the area and are more representative. Community outreach also gives opportunities for health researchers to work in partnership with local government and non-governmental organisations and the potential for ‘researchers’ to better reflect the local demographic. However, this requires time, funding and planning, needing to be resourced and supported from the outset.

It is discouraging that many of the specific requests for training, such as consent where capacity may be an issue, are already free and available across the health research sphere ^52^. This implies more effort is needed to advertise and signpost what training is available. Respondents may simply not have time to search for training, guidelines or toolkits, or it may be that these need to be more specific to a group, illness or setting. Added to this are the low levels of confidence expressed by respondents in their ability to address the needs of the under-served and the call for more information and training on improving the accessibility of study materials, methodologies to promote inclusivity and sharing of best practice. All these areas have previously been put forward to improve representativeness ^7^ and were known and highlighted by the respondents of the survey. It is also disappointing, when considering the previous works, that there is still a lack of focus on the under-served, implying more is required from an organisational perspective. This was particularly noted in those working in mental health and may require further investigation.

The qualitative work suggests that those working in the field of research are aware of the issues surrounding under-served populations. However, there is a sense of exasperation that systems and processes thwart ideas and strategies to increase representation. Highlighted are the processes of grant and funding applications and the ethical review process. These barriers link back to the lack of time and funding to plan research projects, which are specially designed to increase representation. Many state that work in this area takes considerably longer.

The limitations of this work are primarily around the make-up of the respondents. While this work engaged public sector researchers in the UK working with or in the NHS, it does not include the views of large multinational pharmaceutical organisations or private research delivery and monitoring companies. These private sector organisations could be significant in addressing the diversity of research populations. A further limitation is the current climate in the NHS post COVID-19. There was a significant amount of upheaval in the NHS during the pandemic and research activity, outside that of urgent public health, was negatively impacted. That effect is still being felt, with the Department of Health and Social Care setting up a program of work to increase study numbers to pre-pandemic levels under the banner of the Research Reset programme ^53^. The current funding climate and the push to increase the number of studies being carried out means that those working in the field are under significant pressures.

This work suggests that there needs to be a drive from a strategic level to put at the forefront of research the funding and support to engage the under-served. There is room for mandatory targets in recruitment, improved reporting of study populations and strategies utilised to engage those who are under-served in the current research paradigm.

## Conclusion

Lack of inclusivity in research creates problems with the generalisability of findings adding to health disparity and inequality. This survey explored the views of research professionals working in the UK. The UK findings largely concur with those of the USA literature in terms of who are the thought to be the under-served and what factors impact on them being under-served. However, these findings suggest a wider description of minority ethnic and cultural findings in the UK and the inclusion of adolescents and young people as an under-served group. While the concept remains context specific, the barriers to inclusivity included both systemic and local factors. Recommendations are made to engage the under-served prior to the research process, to conduct where possible research in the community and to supply and disseminate information across multiple modalities. Support for researchers to address these recommendations is required in terms of time and funding. A further recommendation is made to explore the use of translation and interpretation in the field of research. There also needs to be a concerted move to address the barriers and support the enablers at an organisational level with additional funding, review of grant application processes, ethical approval and training availability. This work has built on the previous works commissioned by NIHR in the UK. It has drawn out the enablers and barriers to improving inclusivity and made recommendations to further address underrepresentation.

## Data Availability

Discussions have taken place with the Editors of the NIHR Open Research platform. Due to there being small numbers of respondents in some geographical and/or specialist areas, the open data has been limited to the pseudonymised responses from which the enablers and barriers were drawn (see below). This data has been disaggregated from the demographic data to ensure anonymity of respondents. Full anonymised data are available on request to the corresponding author ( Caroline.wroe@nhs.net). Applicants must provide a sound rationale for the use of the data and confirm their commitment to confidentiality. Open Science Framework: Enablers and barriers to engaging under-served groups in research: Survey of the United Kingdom research professional’s views. https://doi.org/10.17605/OSF.IO/F5KNW ^32^ This project contains the following underlying data: Barriers and Enablers raw data.xlsx (Pseudonymised responses from the enablers and barriers section of the questionnaire). Open Science Framework: Enablers and barriers to engaging under-served groups in research: Survey of the United Kingdom research professional’s views. https://doi.org/10.17605/OSF.IO/F5KNW ^32^ This project contains the following extended data: Copy of the survey.pdf Data are available under the terms of the Creative Commons Zero "No rights reserved" data waiver (CC0 1.0 Public domain dedication). Open Science Framework Repository: CROSS checklist for ‘Enablers and barriers to engaging under-served groups in research: Survey of the United Kingdom research professional’s views. https://doi.org/10.17605/OSF.IO/F5KNW
