# Supplementary material for: Enablers and barriers to engaging under-served groups in research: Survey of the United Kingdom research professional’s views
Source: NIHR Open Res. 2023 Dec 18;3:37. Originally published 2023 Jul 26. [Version 2] doi: 10.3310/nihropenres.13434.2 (PMC11319909; doi:10.3310/nihropenres.13434.2)
Supplement: Peer review history

# Reviewer report: Reviewer response for version 2

**DOI:** 10.3310/nihropenres.14675.r30927

**Published:** 2024-02-06

**Authors:** Kelly Beer

**Affiliations:** 1 Murdoch University, Murdoch, Western Australia, Australia; 2 Myositis Discovery Programme, Perron Institute for Neurological and Translational Science, Nedlands, Western Australia, Australia

Thank you very much to the authors for their consideration of the review comments and the subsequent revisions to the article. There are strong improvements to the article resulting from the revisions and enhanced clarity and context, particularly for readers outside of the UK. The article is a great contribution to this field of literature and identifies interesting avenues for future research and understanding to improve equity of access. Thank you for the invitation to review this work.

Is the work clearly and accurately presented and does it cite the current literature?

Yes

If applicable, is the statistical analysis and its interpretation appropriate?

Yes

Are all the source data underlying the results available to ensure full reproducibility?

Partly

Is the study design appropriate and is the work technically sound?

Yes

Are the conclusions drawn adequately supported by the results?

Yes

Are sufficient details of methods and analysis provided to allow replication by others?

Yes

Reviewer Expertise:

Clinical Research Nursing, Research Participation, Research Delivery, Practice Standards

I confirm that I have read this submission and believe that I have an appropriate level of expertise to confirm that it is of an acceptable scientific standard.

# Reviewer report: Reviewer response for version 2

**DOI:** 10.3310/nihropenres.14675.r30926

**Published:** 2024-01-08

**Authors:** Lesley Smith

**Affiliations:** 1 University of Hull, Hull, England, UK

The article has been improved by addressing the comments from peer review and I have no further comments to make.

Is the work clearly and accurately presented and does it cite the current literature?

Yes

If applicable, is the statistical analysis and its interpretation appropriate?

Yes

Are all the source data underlying the results available to ensure full reproducibility?

Yes

Is the study design appropriate and is the work technically sound?

Yes

Are the conclusions drawn adequately supported by the results?

Partly

Are sufficient details of methods and analysis provided to allow replication by others?

Partly

Reviewer Expertise:

Survey design and analysis, mixed-methods research, research with underserved groups.

I confirm that I have read this submission and believe that I have an appropriate level of expertise to confirm that it is of an acceptable scientific standard.

# Reviewer report: Reviewer response for version 1

**DOI:** 10.3310/nihropenres.14571.r30388

**Published:** 2023-10-03

**Authors:** Kelly Beer

**Affiliations:** 1 Murdoch University, Murdoch, Western Australia, Australia; 2 Myositis Discovery Programme, Perron Institute for Neurological and Translational Science, Nedlands, Western Australia, Australia

Thank you for the opportunity to review this article, and congratulations to the authors and study team on their work.

 The article reports on new research conducted to better understand the gaps in access to clinical research within the UK, from the perspective of research professionals. A well-disseminated online survey has been utilised, with a mixed methods approach to elicit information regarding perceived under-served populations, barriers and enablers to participation in clinical research for these populations.

 The article draws on the work within the NIHR INCLUDE program and has strong foundations in recent literature, particularly from within the UK and USA. The article is reported using standard reporting conventions and best practice guides.

 The survey reported is a well-designed tool that has captured views of a large number of health professionals within the NIHR. Overall the article presents an interesting and valuable contribution to this field of knowledge. A number of minor revisions are suggested below to improve flow and readability, as well as to suggest some areas where deeper background or context would further enhance the article.

 It’s always fantastic to read quality research in this important area, and the interest and passion of the authors clearly comes across, particularly in relation to the barriers that research professionals encounter in implementing innovations and strategies to improve equity of access to research.

 The article provides strong evidence that the knowledge and expertise is available to combat many of the barriers to access for under-served groups, however system and resource challenges limit innovation and progress in this space.  

**Minor revisions suggested:**

 Style

- Consider tense used throughout document (particularly within abstract) and keep consistent. 

**Abstract**

 Background

- Suggest consistent use of the term ‘participant’, rather than ‘subject’.
- Correct ‘effects’ to ‘affects’ (‘…and effects clinician prescribing’).
- Rephrase sentence defining under-served to make the relationship between ‘underrepresented’ and ‘under-served’ terms within this study (or within the UK) explicit (i.e. suggest replace ‘thought of’ with ‘defined as’).
- Clarify what is meant by ‘Works..’, i.e. replace with ‘Previous literature’ or similar.
- Suggest replace ‘illness’ with ‘health’.
- Expand sentence regarding survey aim (expand to include what views were being explored).

Methods

- Word-count dependent – could consider including target audience/dissemination information within first sentence.

Results

- Reference here to previous literature, but it is unclear to the reader whether this refers to one previous study or the general evidence in this field.
- Consider wording carefully here – it is a little confusing on first read, as it reads as though the general population were surveyed to identify the groups and factors related to being under-served, but it is important to articulate that this is a reported perception of who is under-served, based on views of research professionals, not a survey of the general population themselves.

Conclusion

- References confirmation of USA findings, however this previous research isn’t referenced otherwise within the abstract. If this article was building on USA research or replicating work from the USA, this should be described in the background section. As per comment for ‘Results’, clarification regarding what previous literature is being confirmed would be helpful. If just one previous study is being referenced/validated, this should be explicit. The second sentence also appears to build on findings from a previous study, but these are interesting stand-alone findings (new knowledge) from this study.
- Again, ensure that it is clear that the study reports on views of research professionals, not views of the under-served populations themselves.
- Last sentence needs to be checked – is there a word missing related to consent? Revise to clarify/correct.

**Keywords**

- These are consistent with the article. Could also use ‘clinical research professional’, ‘clinical research practitioner’, or ‘clinical research nurse’.

**Plain language summary**

- In some sentences the explanatory terms used for simple concepts have increased complexity e.g. ‘…in a questionnaire found on the internet’ could be replaced with ‘…in an online survey’, and ’13 out of 100’ can be replaced with 13%. I would suggest reviewing with lay panel members/consumers, or even utilising AI software that provides ‘plain language’ outputs, to edit this summary and ensure sentences are simple and concise. Ensure the sentence structure clearly identifies the subject (e.g. ‘they’ is used extensively, but it is not always clear who this refers to).
- Unclear as to what is meant by carrying out research ‘…in the community’. Suggest rewording for those unfamiliar with health terminology to be explicit that this means research taking place outside of traditional hospital/research facilities.

**Introduction**

- The first sentence should be referenced and this section would benefit from elaboration regarding the status quo. The lack of diversity in clinical research is the main premise that underpins the study, so it would be interesting here to see some recent statistics around clinical research participation and perhaps an example or two from recent literature as to the impact of including only a homogenous group of participants.
- Line about NIHR INCLUDE – ‘…involving them directly in producing the definition above.’ Which sentence/definition does this refer to? Reword to clarify meaning and retain flow for the reader.
- Consider expanding reference/information regarding the recent work in this area in the USA, as this work is referred to in both the abstract and conclusion.
- Reference in next paragraph first sentence to ‘…nature of the concept…’ – what concept and which ‘terms’ does this refer to? Although it will increase repetition, as a reader it would be much easier to be explicit regarding each sentence’s subject to help the reader understand meaning and intention.  Particularly within this paragraph, but please consider throughout the article. It becomes clearer with second or third read, but is quite confusing on first read.
- When describing how previous works link terms, can you give some examples as well as the descriptors, again to help the reader follow what you are describing? E.g.  ‘Many narrow the focus to engagement within a named group and a specific disease, *e.g. females underrepresented in heart disease research…’*
- Last sentence (related to what the survey covered) – ‘what barriers exist..’, does this refer to barriers for participants or barriers to change?
- The gap in access/involvement for under-served groups is well described, but I would be interested to read a little more within the introduction about any identified gaps in health professional understanding of barriers/enablers that drove this study to investigate their views. I.e. why is understanding health professionals’ views important to improve equity of access and diversity in clinical research?

**Methods**

 Patient and Public Involvement

- It may be appropriate to move the information regarding peer review of the survey (within ‘Survey Development’) to this section, as this constitutes consumer involvement, as meaningful input from the target audience to study design.

Survey development

- It would be helpful to expand upon how the literature review was utilised in the survey design, particularly related to determining the 10 factors that impact being under-served that were used in the survey for ranking.

Recruitment and data collection

- ‘…local clinical research network…’ Needs capitalising?
- Include (CRN) in brackets after ‘Clinical Research Network’ as CRN acronym is used further within the paragraph.
- I don’t think the example provided regarding the North East and North Cumbria CRN is necessary. The dissemination approach is well described and this is a little confusing with the number of email invites listed just for this area. It would be interesting instead to read an estimate of how many research professionals were reached through the network and perhaps an idea of what kind of roles (e.g. research nurse, AHP, etc).
- The questionnaire was described as anonymous, however (optionally) contact details were collected? What was the purpose of the inclusion of contact details and how/what did the study team follow up with for those who provided contact details?

Analysis

- Well described and information provided that would allow replication of methods and analysis.

**Results**

 Demographics

- Did all 945 complete the survey in full? Or was this the total number of completed surveys? Were there many/any who started the survey but did not complete it?
- Could you expand on ‘all components of the NIHR infrastructure and Clinical Specialties…’ for readers who may not be familiar with the NIHR/NHS structure?
- Demographic data is well covered, but this data would be nicely represented as an infographics summary rather than in-text. Particularly the geographical data, also to aid those outside the UK who may not be familiar with regions.

Place of work and role

- Information is provided on the type of role activities (consenting, identifying participants), but was information also collected on the role of respondents? Or length of time in research role?

Who are the under-served

- …in their area’ – Does this refer to geographical area, CRN, or clinical specialism?
- Reference to previous literature could come within Discussion, as it’s not expanded here, so is difficult to know which literature this refers to (a specific study, or the current body of knowledge?).
- Repetition of text within Table 1 descriptor and results text.

What impacts on being under-served

- Repetition of text within Figure 1 descriptor and results text
- Suggest describing the 10 responses to be ranked.

Addressing the needs of the under-served

- Is there a significance of gender to warrant inclusion of that info within the brackets alongside number of respondees? There may be many other sub-group analyses (age, role, region, specialism) that would be relevant to include if this was part of the research question(s), but if there were not specific sub-group analyses done on these questions by gender or any other demographic, I would suggest removing the gender breakdown info to make it easier to read.
- Perhaps consider capitalising the response options to make it easier for the reader to follow which result each sentence is reporting. For example, within the sentence, ‘A significant difference is also seen in those who identify research participants who felt partially equipped….’, it is not clear on first read that the result being reported in this sentence relates to number of respondees identifying as ‘Partially Equipped’, rather than reporting on those who identify research participants.
- Correct to ‘receiving consent’ (rather than ‘taking consent’). Again in the following sentence, rephrase to ‘receiving consent’.

Qualitative analysis

 Enablers and Barriers

- May be helpful to firstly introduce/summarise the main themes found in each sub-section before expanding on each.
- An overview/summary table with the themes identified across Enablers and Barriers could work well?
- There is some cross-over here with content that could easily fit within the Discussion section or is repeated there.
- For consistency, include sub-group analysis for barriers, to report on most cited barriers for primary care and mental health groups.

Further information and training

- The final paragraph to this section could fit within the Discussion, or even Conclusion, as the findings are nicely summarised here.

**Discussion**

- Provides a nice summary and delve into the results, with interesting observations drawn and areas for further research identified.
- Do draw the reader back to the context of the study to acknowledge limitations – this study is reporting on perceived factors that impact being under-served from the perspective of research professionals, not based on surveying the general population or under-served populations themselves.

**Conclusion**

- I would prefer a high level summary of the findings from this study regarding who is under-served and the main factors related to this, rather than the reference back to the USA literature.
- Review phrase ‘…to conduct where possible research in the community…’. Might need a comma or just a tweak.
- Final sentence refers back to ‘underrepresentation’. For consistency, should this be amended to refer to the under-served populations?

Is the work clearly and accurately presented and does it cite the current literature?

Yes

If applicable, is the statistical analysis and its interpretation appropriate?

Yes

Are all the source data underlying the results available to ensure full reproducibility?

Partly

Is the study design appropriate and is the work technically sound?

Yes

Are the conclusions drawn adequately supported by the results?

Yes

Are sufficient details of methods and analysis provided to allow replication by others?

Yes

Reviewer Expertise:

Clinical Research Nursing, Research Participation, Research Delivery, Practice Standards

I confirm that I have read this submission and believe that I have an appropriate level of expertise to confirm that it is of an acceptable scientific standard, however I have significant reservations, as outlined above.

# Response

**Published:** 2023-11-28

**Authors:** Dorothy Coe

**Affiliations:** North East North Cumbria LCRN, Newcastle Upon Tyne Hospitals NHS Foundation Trust, Newcastle upon Tyne, England, UK

*Response: Thank you for the detailed review of our work. We have discussed the comments made and revised in light of our discussions.*

 Style: Consider tense used throughout document (particularly within abstract) and keep consistent.

*Response: Reviewed as requested.*

 Abstract:

**Background**

 Suggest consistent use of the term ‘participant’, rather than ‘subject’.

 Correct ‘effects’ to ‘affects’ (‘…and effects clinician prescribing’).

 Rephrase sentence defining under-served to make the relationship between ‘underrepresented’ and ‘under-served’ terms within this study (or within the UK) explicit (i.e. suggest replace ‘thought of’ with ‘defined as’).

 Clarify what is meant by ‘Works..’, i.e. replace with ‘Previous literature’ or similar.

 Suggest replace ‘illness’ with ‘health’.

 Expand sentence regarding survey aim (expand to include what views were being explored).

**Methods**

 Word-count dependent – could consider including target audience/dissemination information within first sentence.

**Results**

 Reference here to previous literature, but it is unclear to the reader whether this refers to one previous study or the general evidence in this field.

 Consider wording carefully here – it is a little confusing on first read, as it reads as though the general population were surveyed to identify the groups and factors related to being under-served, but it is important to articulate that this is a reported perception of who is under-served, based on views of research professionals, not a survey of the general population themselves.

**Conclusion**

 References confirmation of USA findings, however this previous research isn’t referenced otherwise within the abstract. If this article was building on USA research or replicating work from the USA, this should be described in the background section. As per comment for ‘Results’, clarification regarding what previous literature is being confirmed would be helpful. If just one previous study is being referenced/validated, this should be explicit. The second sentence also appears to build on findings from a previous study, but these are interesting stand-alone findings (new knowledge) from this study.

 Again, ensure that it is clear that the study reports on views of research professionals, not views of the under-served populations themselves.

 Last sentence needs to be checked – is there a word missing related to consent? Revise to clarify/correct.

*Response: A new abstract as been produced taking into account the above comments and the allowed word count.*

 Keywords:

 These are consistent with the article. Could also use ‘clinical research professional’, ‘clinical research practitioner’, or ‘clinical research nurse’.

*Response: These terms are not consistent with our work as participants included non-clinical research staff.*

 Plain language summary:

 In some sentences the explanatory terms used for simple concepts have increased complexity e.g. ‘…in a questionnaire found on the internet’ could be replaced with ‘…in an online survey’, and ’13 out of 100’ can be replaced with 13%. I would suggest reviewing with lay panel members/consumers, or even utilising AI software that provides ‘plain language’ outputs, to edit this summary and ensure sentences are simple and concise. Ensure the sentence structure clearly identifies the subject (e.g. ‘they’ is used extensively, but it is not always clear who this refers to).

 Unclear as to what is meant by carrying out research ‘…in the community’. Suggest rewording for those unfamiliar with health terminology to be explicit that this means research taking place outside of traditional hospital/research facilities.

*Response: Advice taken as suggested and reviews made in light of comments received.*

 Introduction:

 The first sentence should be referenced and this section would benefit from elaboration regarding the status quo. The lack of diversity in clinical research is the main premise that underpins the study, so it would be interesting here to see some recent statistics around clinical research participation and perhaps an example or two from recent literature as to the impact of including only a homogenous group of participants.

 Line about NIHR INCLUDE – ‘…involving them directly in producing the definition above.’ Which sentence/definition does this refer to? Reword to clarify meaning and retain flow for the reader.

 Consider expanding reference/information regarding the recent work in this area in the USA, as this work is referred to in both the abstract and conclusion.

 Reference in next paragraph first sentence to ‘…nature of the concept…’ – what concept and which ‘terms’ does this refer to? Although it will increase repetition, as a reader it would be much easier to be explicit regarding each sentence’s subject to help the reader understand meaning and intention.  Particularly within this paragraph, but please consider throughout the article. It becomes clearer with second or third read, but is quite confusing on first read.

 When describing how previous works link terms, can you give some examples as well as the descriptors, again to help the reader follow what you are describing? E.g.  ‘Many narrow the focus to engagement within a named group and a specific disease, e.g. females underrepresented in heart disease research…’

 Last sentence (related to what the survey covered) – ‘what barriers exist..’, does this refer to barriers for participants or barriers to change?

 The gap in access/involvement for under-served groups is well described, but I would be interested to read a little more within the introduction about any identified gaps in health professional understanding of barriers/enablers that drove this study to investigate their views. I.e. why is understanding health professionals’ views important to improve equity of access and diversity in clinical research?

*Response: Revised with comments taken into account.*

 Methods:

 Patient and Public Involvement

 It may be appropriate to move the information regarding peer review of the survey (within ‘Survey Development’) to this section, as this constitutes consumer involvement, as meaningful input from the target audience to study design.

 Survey development

 It would be helpful to expand upon how the literature review was utilised in the survey design, particularly related to determining the 10 factors that impact being under-served that were used in the survey for ranking.

 Recruitment and data collection

 ‘…local clinical research network…’ Needs capitalising?

 Include (CRN) in brackets after ‘Clinical Research Network’ as CRN acronym is used further within the paragraph.

 I don’t think the example provided regarding the North East and North Cumbria CRN is necessary. The dissemination approach is well described and this is a little confusing with the number of email invites listed just for this area. It would be interesting instead to read an estimate of how many research professionals were reached through the network and perhaps an idea of what kind of roles (e.g. research nurse, AHP, etc).

 The questionnaire was described as anonymous, however (optionally) contact details were collected? What was the purpose of the inclusion of contact details and how/what did the study team follow up with for those who provided contact details?

 Analysis

 Well described and information provided that would allow replication of methods and analysis.

*Response: PPIE moved as suggested although the Editors may have an alternative suggestion. The example from the NENC CRN was requested by the editors. Granular information on job type (Nurse AHP etc) was not collected. Thank you for pointing out the inaccuracy regarding anonymity. The survey was anonymous. Respondents were invited to leave their contact details for the NIHR under-served programme board. This was to collect 'best practice' examples and was outside of the research project reported. The study staff were not involved in the collection of email addresses or the production of best practice examples.*

 Results:

 Demographics

 Did all 945 complete the survey in full? Or was this the total number of completed surveys? Were there many/any who started the survey but did not complete it?

 Could you expand on ‘all components of the NIHR infrastructure and Clinical Specialties…’ for readers who may not be familiar with the NIHR/NHS structure?

 Demographic data is well covered, but this data would be nicely represented as an infographics summary rather than in-text. Particularly the geographical data, also to aid those outside the UK who may not be familiar with regions.

 Place of work and role

 Information is provided on the type of role activities (consenting, identifying participants), but was information also collected on the role of respondents? Or length of time in research role?

 Who are the under-served…in their area’ – Does this refer to geographical area, CRN, or clinical specialism?

 Reference to previous literature could come within Discussion, as it’s not expanded here, so is difficult to know which literature this refers to (a specific study, or the current body of knowledge?).

 Repetition of text within Table 1 descriptor and results text.

 What impacts on being under-served

 Repetition of text within Figure 1 descriptor and results text

 Suggest describing the 10 responses to be ranked.

 Addressing the needs of the under-served

 Is there a significance of gender to warrant inclusion of that info within the brackets alongside number of respondees? There may be many other sub-group analyses (age, role, region, specialism) that would be relevant to include if this was part of the research question(s), but if there were not specific sub-group analyses done on these questions by gender or any other demographic, I would suggest removing the gender breakdown info to make it easier to read.

 Perhaps consider capitalising the response options to make it easier for the reader to follow which result each sentence is reporting. For example, within the sentence, ‘A significant difference is also seen in those who identify research participants who felt partially equipped….’, it is not clear on first read that the result being reported in this sentence relates to number of respondees identifying as ‘Partially Equipped’, rather than reporting on those who identify research participants.

 Correct to ‘receiving consent’ (rather than ‘taking consent’). Again in the following sentence, rephrase to ‘receiving consent’.

 Qualitative analysis

 Enablers and Barriers

 May be helpful to firstly introduce/summarise the main themes found in each sub-section before expanding on each.

 An overview/summary table with the themes identified across Enablers and Barriers could work well?

 There is some cross-over here with content that could easily fit within the Discussion section or is repeated there.

 For consistency, include sub-group analysis for barriers, to report on most cited barriers for primary care and mental health groups.

 Further information and training

 The final paragraph to this section could fit within the Discussion, or even Conclusion, as the findings are nicely summarised here.

*Response: The respondent interpreted 'in their area'. Repetition, the submission requires tables and figures are discussed within the text, therefore to some extent repetition cannot be avoided. However a formatting error has been corrected which had led to whole paragraphs being repeated. The 10 factors are described within the figure. The inclusion of gender (whether significant or not) was requested by the editors. We feel that additional capitisation detracts from the presentation of the work, but will be guided by the editors. Consent phrasing has be revised as suggested. We feel an overview/summary table in not required and would lead to repetition. Sub-group analysis has been added to the 'further information and training' section. We have chosen to leave the interim conclusions at the end of the results section.*

 Discussion:

 Provides a nice summary and delve into the results, with interesting observations drawn and areas for further research identified.

 Do draw the reader back to the context of the study to acknowledge limitations – this study is reporting on perceived factors that impact being under-served from the perspective of research professionals, not based on surveying the general population or under-served populations themselves.

*Response: Additions to the limitations section have been added.*

 Conclusion:

 I would prefer a high level summary of the findings from this study regarding who is under-served and the main factors related to this, rather than the reference back to the USA literature.

 Review phrase ‘…to conduct where possible research in the community…’. Might need a comma or just a tweak.

 Final sentence refers back to ‘underrepresentation’. For consistency, should this be amended to refer to the under-served populations?

*Response: A higher level summary has been added as requested. Thank you for pointing out errors in phrasing.*

# Reviewer report: Reviewer response for version 1

**DOI:** 10.3310/nihropenres.14571.r30386

**Published:** 2023-09-29

**Authors:** Lesley Smith

**Affiliations:** 1 University of Hull, Hull, England, UK

The research article describes the results of a large survey of research professionals' views of engaging individuals who are under represented in health research studies in the UK. This is an important topic and this manuscript adds value to the existing literature on the topic.

 It is well written with a clear structure. The methods are transparent and comprehensively reported with the exception of the qualitative analysis which need expanding. A description of how the barriers and enablers were derived and categorised from the themes would enhance the methods.

 The interpretation of the following statement needs more cautious interpretation. "Those who consent research participants were more likely to feel partially equipped to address the needs of the under-served compared to those who do not consent (p=0.013), suggesting that face to face contact improves confidence in engaging the under-served."

 Causality cannot be inferred about engagement leading to confidence from this cross-sectional survey. Reverse causality cannot be ruled out whereby those that are more confident engage in recruitment more. All you can say is that there was an association, not that it suggests a cause effect relationship.

Is the work clearly and accurately presented and does it cite the current literature?

Yes

If applicable, is the statistical analysis and its interpretation appropriate?

Yes

Are all the source data underlying the results available to ensure full reproducibility?

Yes

Is the study design appropriate and is the work technically sound?

Yes

Are the conclusions drawn adequately supported by the results?

Partly

Are sufficient details of methods and analysis provided to allow replication by others?

Partly

Reviewer Expertise:

Survey design and analysis, mixed-methods research, research with underserved groups.

I confirm that I have read this submission and believe that I have an appropriate level of expertise to confirm that it is of an acceptable scientific standard, however I have significant reservations, as outlined above.

# Response

**Published:** 2023-11-28

**Authors:** Dorothy Coe

**Affiliations:** North East North Cumbria LCRN, Newcastle Upon Tyne Hospitals NHS Foundation Trust, Newcastle upon Tyne, England, UK

Thank you. We have drawn back on our conclusion as per your suggestion.
